# Nonrigid Image Registration in Digital Subtraction Angiography Using Multilevel B-Spline

**DOI:** 10.1155/2013/236315

**Published:** 2013-07-22

**Authors:** Mansour Nejati, Saeid Sadri, Rassoul Amirfattahi

**Affiliations:** Digital Signal Processing Research Laboratory, Department of Electrical and Computer Engineering, Isfahan University of Technology, Isfahan 84156-83111, Iran

## Abstract

We address the problem of motion artifact reduction in digital subtraction angiography (DSA) using image registration techniques. Most of registration algorithms proposed for application in DSA, have been designed for peripheral and cerebral angiography images in which we mainly deal with global rigid motions. These algorithms did not yield good results when applied to coronary angiography images because of complex nonrigid motions that exist in this type of angiography images. Multiresolution and iterative algorithms are proposed to cope with this problem, but these algorithms are associated with high computational cost which makes them not acceptable for real-time clinical applications. In this paper we propose a nonrigid image registration algorithm for coronary angiography images that is significantly faster than multiresolution and iterative blocking methods and outperforms competing algorithms evaluated on the same data sets. This algorithm is based on a sparse set of matched feature point pairs and the elastic registration is performed by means of multilevel B-spline image warping. Experimental results with several clinical data sets demonstrate the effectiveness of our approach.

## 1. Introduction

Digital subtraction angiography (DSA) is a widely used fluoroscopy technique for vascular imaging [1, 2]. This technique produces a sequence of projection X-ray images of blood vessels that is used in diagnosis and treatment. The first few images of the sequence are precontrast or mask images taken prior to the injection of the contrast medium, and thus, vessels are not visible in them. Other images in the sequence that are acquired during the passage of the contrast medium are often referred to as the contrast or live images. By temporal subtraction of a mask image from the each live image, overlying structure besides the blood vessels in the live images is largely cancelled and visualization of vessels is improved. However, mask and live images are acquired at different times, and voluntary or involuntary patient motions during the acquisition procedure may produce significant motion artifacts that would appear in the resulting DSA images. Such artifacts may hamper proper interpretation of the images and even lead to misdiagnosis [2]. To cope with this problem, aligning of the mask and live images is required prior to subtracting the images.

Image registration refers to the process of spatially aligning two or more images of the same scene taken at different times, and from different viewpoints [3]. Image registration has been an active area of research in different domains and applications, in the last few decades. Survey and categorization of image registration methods may be found in papers by Brown [4], van den Elsen et al. [5], Maintz and Viergever [6], and Zitová and Flusser [3]. A collection of papers reviewing methods particularly suitable for registration of medical images has been edited into a book by Hajnal et al. [7] and a detailed and extensive framework of DSA image registration has been provided in [2].

During the past twenty years, many efforts have been made to reduce the motion artifacts in DSA images using image registration algorithms [2, 8–21]. Most of these algorithms have designed for application in peripheral and cerebral angiography in which we mainly deal with global rigid motions. In this case, rigid and affine registration methods can be used for aligning the mask and live images. However, the motions in coronary angiography images are much more complex. The nonrigid motion of the tissues in coronary angiography images such as motion induced by heart beating and breathing is complicated and, thus, a simple translation or rotation of the misregistered image cannot eliminate the artifacts caused by these motions. In order to be able to correct for such complex motions, registration techniques should be designed so as to have local control.

To cope with local differences between the mask and live images in coronary angiography, a few algorithms have been proposed in past decade. Yang et al. [20] proposed a multiresolution registration algorithm in which the mask image is decomposed to coarse and fine subimage blocks iteratively and each block is rigidly registered to the live image. Wang and Zhang [19] proposed an iterative refinement algorithm for the registration in DSA. In their method, nonrigid motions are iteratively modeled using thin-plate spline (TPS) which is calculated from a set of corresponding interest points between two images. Iterative nature of these registration algorithms leads to high computational time which makes them not acceptable for real-time clinical applications.

The purpose of this paper is to develop an effective and fast algorithm for nonrigid image registration in digital subtraction angiography. In this algorithm, a proposed approach based on an edge-detection scheme [2, 15], and Harris corner detector [22], is used for the feature-based selection of control points on the live image. The displacement of selected control points is calculated by means of template matching in which a multiple initialized hill-climbing approach is used for the optimization of similarity measure. For comparison of different similarity measures, an objective measurement method is developed in this paper on simulated data. Based on the comparison of the results, entropy of histogram of differences (ENT) is selected as a suitable similarity measure for registration in DSA. The final correction in proposed registration method is performed by warping the mask image using multilevel B-spline interpolation function [23]. However, prior to image warping, morphological operators are applied to the mask and live images to reduce the gray-level distortion artifacts in background of resulting subtraction image.

The rest of this paper is organized as follows. The proposed registration approach is described in detail in Section 2. In addition, evaluation method of similarity measures is described in this section. An overview of the algorithm is presented in Section 3. Results of experiments on simulated and real clinical angiographic image data sets and the related conclusions are given in Sections 4 and 5, respectively.

## 2. Registration Approach

In our approach, the registration procedure is carried out in three main steps. In the first step, a set of control points *P* = {**p**_*i*_} is automatically selected from the live image by using a proposed hybrid approach based on an edge-detection scheme and Harris corner detector. In the second step, the displacements of the selected control points **p**_*i*_ ∈ *P* are computed by means of block matching in which the entropy of histogram of differences is exploited as similarity measure and hill-climbing approach with multiple initial points is used for the optimization. Multilevel B-spline interpolation is then used to smoothly construct the complete displacement vector field based on estimated displacements of control points. Finally, motion correction is performed according to this displacement vector field by elastic warping of the mask image with respect to the live image. However to reduce the gray-level distortion artifacts in background of DSA image resulted after registration, grayscale morphology is applied to the mask and live images prior to image warping.

### 2.1. Control Points Selection

For registration of two images, computing a dense pixel correspondence between them is required. A common approach to obtain a dense correspondence is to merely calculate the correspondence for a selected set of so-called control points from which the overall correspondence can then be estimated by a proper interpolation or approximation.

In the easiest way, control points can be selected on a regular grid, but more appropriate method is the selection based on image features. In the particular case of DSA images, major artifacts only appear in those regions of mask and live images where strong object edges are present and are not coincided [2, 15]. Since the edges can be matched better than homogeneous regions, strong edges in the image would be proper image features for selection of control points. Control points can also be selected by performing an interest point detector such as Harris corner detector. Harris corner detector locates the corners by detecting the locally unique image neighborhoods. The centers of such neighborhoods can be more robustly matched than points located merely on a strong edge. Therefore, we used a hybrid approach that takes the advantages of both important edges and Harris corner detector for selection of control points. The following is a summary of the steps to be followed in the proposed control points selection algorithm with a minimum distance, *D*_min⁡_, with respect to each other.

(1) Compute the gradient magnitude of the live image based on an edge-detection filtering with the application of Gaussian derivative [24] at scale *σ* = 1 (standard deviation of Gaussian). Then, normalize the gradient magnitude values to [0 1].
(2) Locate the strong edges by thresholding the gradient magnitude image at a value *t*_0_. Pixels with gradient magnitude values less than *t*_0_ are considered pixels belonging to background and their values are put to 0 and values of rest of pixels are kept unchanged. Let *G* indicates the obtained image in step 2.
(3) Calculate the Harris corner response *R* of the live image as follows:
  (1) $$\begin{array}{c} R={\hat{I}}_{x}^{2}\cdot {\hat{I}}_{y}^{2}-{\hat{I}}_{xy}^{2}-k{\left({\hat{I}}_{x}^{2}+{\hat{I}}_{y}^{2}\right)}^{2}, \end{array}$$
 with
  (2) $$\begin{array}{c} {\hat{I}}_{x}=I_{x}*g,\\ {\hat{I}}_{y}=I_{y}*g,\\ {\hat{I}}_{xy}=\left(I_{x}\cdot I_{y}\right)*g, \end{array}$$
 where *I*_*x*_ and *I*_*y*_ are partial derivatives of image *I* in *x* and *y*, respectively, *g* is a circular Gaussian window, and ∗ denotes the convolution operator. The value of sensitivity parameter *k* has to be determined empirically and in this paper is set to 0.12.
(4) Set negative values of *R* to 0 and normalize nonnegative values to the interval [0 1].
(5) Calculate weighted average $\hat{R}=\alpha G+\beta R$ for *α* = 0.3 and *β* = 0.7.
(6) Find local maxima greater than *t*_1_ = 0.1 in $\hat{R}$ by examining of circular neighborhoods of radius *r* = 5, sort them according to $\hat{R}$ from the largest to the smallest, and consider them as candidate control points.
(7) Starting from the candidate control point with the largest $\hat{R}$, move candidates to set of control points *P* = {**p**_*i*_} one at a time. After moving a candidate to *P*, remove all candidates that are within distance *D*_min⁡_ of it. Repeat the process until no more candidates remain.

**Figure 1:**
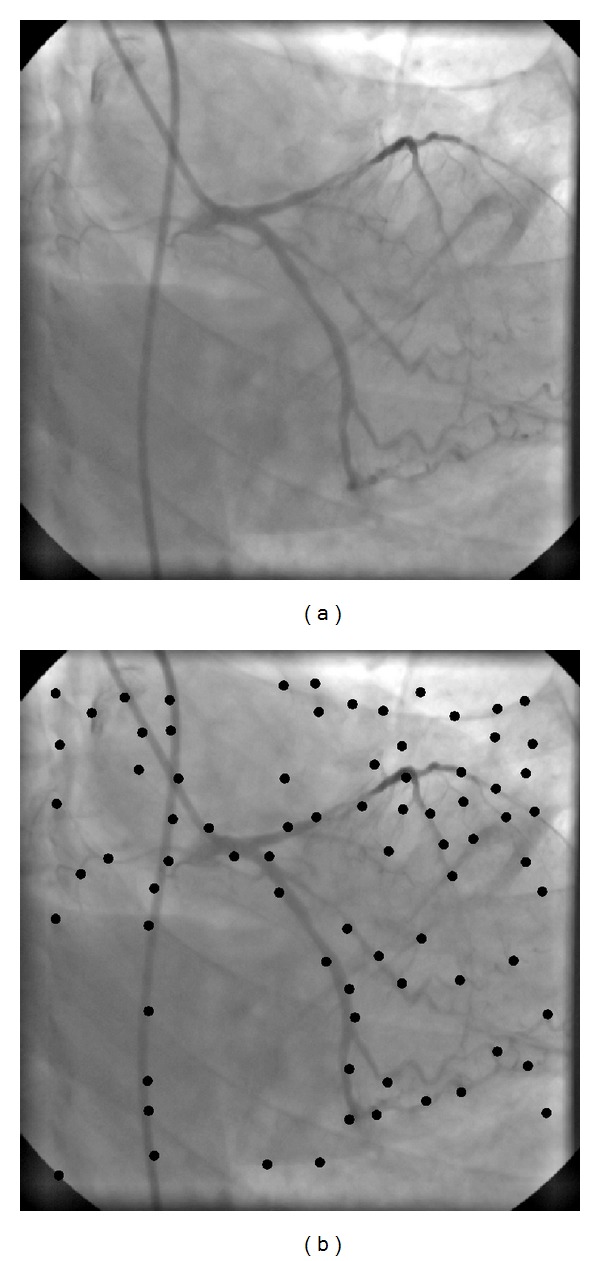
Example of control points selection. (a) A live image from a 512 × 512 × 40 coronary angiographic image sequence. (b) Selected control points (black dots) by using the proposed feature-based approach.

The parameter *t*_0_ in step 2 of above procedure is equal to the average of computed gradient magnitude values. The values of other parameters have been selected empirically upon the images under test (angiographic images of size 512 × 512 with gray-value resolution of 8 bits, i.e., 256 gray levels). An example of a control point selection based on this approach is shown in Figure 1.

### 2.2. Control Points Displacements Calculation

After selection of control points **p**_*i*_ = (*x*_*i*_, *y*_*i*_), *i* = 1,2,…, *n* in the live image, this second step consists of calculating the displacement of each control point, namely, searching for the corresponding points **q**_*i*_ = (*x*_*i*_′, *y*_*i*_′), *i* = 1,2,…, *n* in the mask image. There are two major categories for calculation of local motion: optic-flow techniques and the template-matching based techniques. As discussed in [2] the basic assumptions of optic-flow techniques do not apply to digital X-ray projection imaging. In addition, these techniques are sensitive to the inward flow of injected contrast medium and experiments carried out by Meijering et al. [2], have demonstrated that the optic-flow technique will not yield accurate results when applied to these images. Thus, we employ template-matching based techniques, which can be made more robust against the local dissimilarities caused by inward flow of contrast in angiographic images, by a proper choice for the similarity measure. Then the similarity measure comes to be the crucial factor of the whole matching process.

Several similarity measures have been introduced and applied to register images. In our application, a similarity measure with robustness against the mean gray-level offsets and local dissimilarities caused by contrasted vessels is required. In order to choose a suitable similarity measure for registration in DSA, an objective measurement method is developed in this paper on simulated data that makes the comparison of similarity measures possible. 

The most commonly used similarity measures which have been used during the past fifteen years for registration in DSA include energy of histogram of differences (EHD) [2, 12, 13], correlation coefficient (CC) [14], mutual information (MI) [16, 21], and structural similarity index (SSIM) [19]. In our registration algorithm, we employed the entropy of normalized histogram of difference image (ENT) as similarity measure to find corresponding control points. The ENT was first used as a measure of similarity in template-matching procedure by Buzug et al. [11]. In the following, we demonstrate that ENT is more suitable similarity measure for application of registration in DSA than four other ones.

#### 2.2.1. Comparison of Similarity Measures

A method that can be used for quantitative evaluation of similarity measures is using them for determining the correspondences between two images and then calculating the errors. It is obvious that calculation of the errors requires knowledge about the true correspondences, whereas there is no any ground truth for the angiographic images. To obtain a mask and a live image with nonlinear geometric difference and known correspondences, we developed a simple approach for generating a mask image from a live image and then applied a geometric transformation with known parameters to the live image.

**Figure 2:**
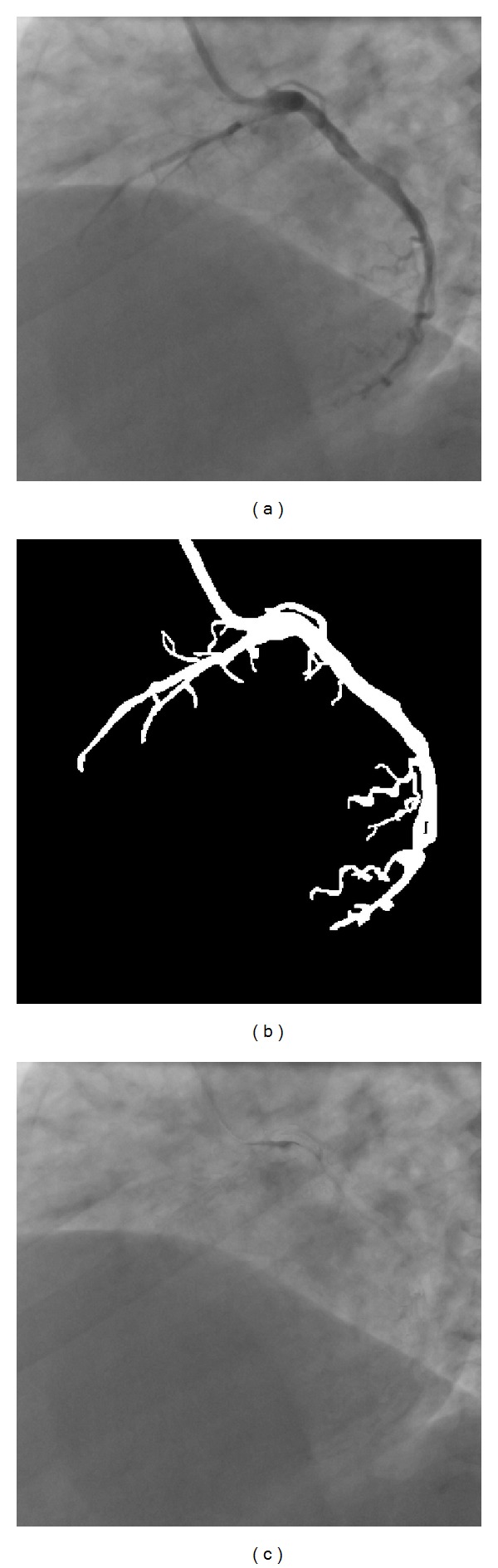
Example of mask image generation from a live image by using the proposed approach. (a) A live image from a coronary angiographic image sequence. (b) Result of vessel segmentation. (c) Generated mask image.

To generate a mask image from a live image, we employed a semiautomatic approach. In this approach, a cine angiography in which anatomical structures such as ribs, spine, diaphragm, and other visible tissues but vessels have very small movements (in ideal case, do not have any movement) is needed. Let *K* denote the number of images (frames) in this cine angiography where each image is defined by the function *f*(*x*, *y*, *t*), *t* = 1,2,…, *K* and let *L*(*x*, *y*) = *f*(*x*, *y*, *k*) denote a live image in this sequence. For generating a mask image *M* from live image, at first the visible vessels in the live image are manually segmented thereupon a binary image *B* is obtained in which pixels belonging to vessels are labeled 1 and other pixels are labeled 0. According to the result of segmentation, generation of mask image *M* from live image *L* is done as follows:

(3) M(x,y)={L(x,y),if  B(x,y)=0,max⁡(f(x,y,t)), t=k−δ,…,k+δ,if  B(x,y)=1,

where *k* and *δ* may be selected experimentally such that the best result is achieved.  Figure 2 gives a mask generation example by means of the above approach for *k* = 15 and *δ* = 5. Figures 2(a), 2(b), and 2(c), respectively, show a live image, result of vessel segmentation, and generated mask image from the live image.

After generating the mask image from a live image, a set of control points are selected in the live image using control point selection method described in Section 2.1, which can be represented as (*x*_*i*_, *y*_*i*_), where *i* = 1,2,…, *n*, and *n* is the number of control points. For each control point, an uncorrelated random shift to both *x* and *y* dimensions ranging from −10 to 10 pixels is added. A new set of random control points are thus formed, which can be written as (*x*_*i*_ + *dx*_*i*_, *y*_*i*_ + *dy*_*i*_), *i* = 1,2,…, *n*. With the generated control point pairs, a new simulated live image is produced by warping of it using multilevel B-spline interpolation. Then, for each of control points in simulated live image located at (*x*_*i*_ + *dx*_*i*_, *y*_*i*_ + *dy*_*i*_), a template of *w* × *w* pixel is taken around it and corresponding control point in the generated mask image is searched by template-matching technique with a certain similarity measure.

We know the coordinates (*x*_*i*_, *y*_*i*_), *i* = 1,2,…, *n*, of the true corresponding control points in the mask image. Therefore, by calculating the RMS error between the true control points (*x*_*i*_, *y*_*i*_) and those control points determined by template matching (*x*_*i*_′, *y*_*i*_′), *i* = 1,2,…, *n*, the similarity measures can be evaluated quantitatively. The RMS error, *δ*_RMS_, is computed as

(4) $$\begin{array}{c} \delta_{\text{RMS}}=\sqrt{\frac{1}{n}\left(\sum_{i=1}^{n}{||\left(x_{i},y_{i})-(x_{i}^{\prime },y_{i}^{\prime }\right)||}^{2}\right)}. \end{array}$$

**Figure 3:**
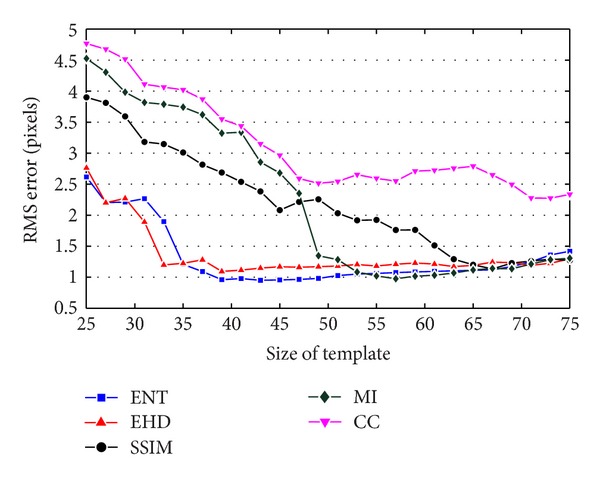
Evaluation of different similarity measure based on RMS error obtained in determination of corresponding control points between live and mask images.

We carried out this experiment for five similarity measures including EHD, CC, MI, SSIM, and ENT with different sizes of template ranging from *w* = 25 to *w* = 75 and for each size, ten times. Finally, average of RMS errors for each similarity measure was calculated.  Figure 3 shows the RMS error curves with respect to template size. As can be seen from Figure 3, the entropy of histogram of differences (ENT) leads to least RMS error for template sizes ranging from 39 to 53. On the other hand, the computational complexity of ENT is much lower than MI and SSIM. Hence, in our registration algorithm we select the ENT as a suitable similarity measure.

#### 2.2.2. Optimization

After automatic selection of control points in the live image, the displacement vector of each control point with respect to mask image is determined in second step of registration process by means of template-matching technique by using entropy-based similarity measure. Given a certain similarity measure, the optimal displacement vector according to this measure is the vector **d** = (*dx*, *dy*) for which the similarity measure gets a global extreme value. Finding this extreme is equivalent to the function optimization problem, for which a large number of algorithms exist. The most straightforward approach is to impose constraints on the maximum acceptable displacement in both *x*- and *y*-direction and to perform a full search, that is, to evaluate the similarity measure for every possible displacement, subject to the constraints |*dx* | ≤*dx*_max⁡_ and |*dy* | ≤*dy*_max⁡_ [25].

Utilizing of an exhaustive search procedure in template matching is computationally too expensive, particularly when the maximum possible displacements of control points, *dx*_max⁡_ and *dy*_max⁡_, are large. Therefore, a more efficient strategy is needed. This efficiency is considered from two aspects: first, the optimization method must be robust and reliable in order to obtain accurate correspondences. Second, it must have low computational cost since we have to reduce computation time to a clinically acceptable level. In the present paper, the hill-climbing algorithm which is a simplification of well-known Powell's direction set method [26] is employed for optimization.

Finding the global optimum of a function cannot be guaranteed by most of optimization techniques and they may be trapped into local optima. Thus, if resulting match surface associated to a certain similarity measure has a pronounced global optimum but in addition shows many local optima, hill climbing is very likely to trap in a local optimum. The behavior of a similarity measure is strongly related to the size of the windows in which it is computed [25]. Buzug and Weese [13] reported that with histogram-based similarity measures such as EHD and ENT, a window size of about 50 × 50 pixels leads to smooth match surfaces, which allows for optimization by means of a simple and fast approach such as hill climbing. Nevertheless, the trapping into local optima is still probable. One effective algorithm to increase the reliability is performing hillclimbing iteratively, each time with a different initial point, and then keeping the best solution. Assuming that maximum displacement of control points in both *x*- and *y*-direction would not exceed from 20 pixels, in our registration algorithm the hillclimbing is performed four times iteratively with initial points **d**_1_ = (5,5), **d**_2_ = (5, −5), **d**_3_ = (−5,5), and **d**_4_ = (−5, −5). These initial points were selected experimentally.

### 2.3. Image Warping

The final correction in registration process is performed by warping the mask image with respect to the live image. In order to be able to carry out the warping of the mask image, it is required to have a complete description of the displacement vector field. That is, the displacement **d** must be known for every point in the image. This displacement vector field can be evaluated through interpolation of displacements **d**_*i*_ = (*dx*_*i*_, *dy*_*i*_) of control points **p**_*i*_ = (*x*_*i*_, *y*_*i*_), *i* = 1,2,…, *n*. If we rearrange the control points and corresponding displacements as follows:

(5) $$\begin{array}{c} \left\{\left(x_{i},y_{i},dx_{i}\right):i=1,2,\ldots ,n\right\}, \end{array}$$

(6) $$\begin{array}{c} \left\{\left(x_{i},y_{i},dy_{i}\right):i=1,2,\ldots ,n\right\} \end{array}$$

we need to find two single-valued functions *f*_*X*_(*x*, *y*) and *f*_*Y*_(*x*, *y*) that interpolate scattered data points given in (5) and (6), respectively. That is, these functions satisfy

(7) $$\begin{array}{c} dx_{i}=f_{X}\left(x_{i},y_{i}\right),\;i=1,2,\ldots ,n,\\ dy_{i}=f_{Y}\left(x_{i},y_{i}\right),\;i=1,2,\ldots ,n. \end{array}$$

In this paper, image warping is performed using multilevel B-spline interpolation function [23]. Multilevel B-splines can be used for smooth interpolation or approximation of irregularly spaced samples. With respect to the thin-plate spline (TPS) interpolation [27, 28] which is one of the most widely used method in the registration of images with nonlinear distortions, multilevel B-spline interpolation has two major advantages: it has lower computational complexity, and because of local support of B-spline basis functions as opposed to TPS, we can manipulate the local deformations better than TPS. In the next section, a brief overview of multilevel B-spline interpolation of scattered bivariate data is presented.

#### 2.3.1. Multilevel B-Spline Interpolation

In multilevel B-spline interpolation algorithm, a multiresolution approach is formulated to compute an interpolating surface from a set of scattered data points. Let *Ω* = {(*x*, *y*) | 0 ≤ *x* < *m*, 0 ≤ *y* < *n*} be a rectangular domain in the *xy*-plane. Consider a set of scattered data points **P** = {(*x*_*c*_, *y*_*c*_, *z*_*c*_)}, where (*x*_*c*_, *y*_*c*_) is a point in *Ω* and *z*_*c*_ denotes its height (in registration algorithm, (*x*_*c*_, *y*_*c*_) is coordinate of a control point extracted from live image, and *z*_*c*_ is the calculated displacement of it with respect to the mask image in *x*- or *y*-direction). Let Φ be a (*m* + 3)×(*n* + 3) lattice of control points overlaid on domain *Ω* and let *ϕ*_*ij*_ be the value of the *ij*th control point on lattice Φ located at (*i*, *j*) for *i* = −1,0,…, *m* + 1 and *j* = −1,0,…, *n* + 1. Note that, the control points discussed in previous sections are feature points extracted from an image, but the control points *ϕ*_*ij*_ are the points that control the behavior of B-spline approximation function *f* which is defined in terms of these control points by

(8) $$\begin{array}{c} f\left(x,y\right)=\sum_{k=0}^{3}\;\sum_{l=0}^{3}B_{k}\left(s\right)B_{l}\left(t\right)\phi_{\left(i+k)(j+l\right)}, \end{array}$$

where *i* = ⌊*x*⌋ − 1, *j* = ⌊*y*⌋ − 1, *s* = *x* − ⌊*x*⌋, and *t* = *y* − ⌊*y*⌋. *B*_*k*_ and *B*_*l*_ are uniform cubic B-spline basis functions defined as

(9) B0(t)=(1−t)36,B1(t)=3t3−6t2+46,B2(t)=−3t3+3t2+3t+16,B3(t)=t36,

where 0 ≤ *t* < 1. To obtain the function *f*, we must find the values of *ϕ*_*ij*_ in Φ that best approximate the scattered data in **P**. To determine the unknown control lattice Φ, first consider one data point (*x*_*c*_, *y*_*c*_, *z*_*c*_) in **P**. The function value *f*(*x*_*c*_, *y*_*c*_) relates to the sixteen control points *ϕ*_*ij*_ in the neighborhood of (*x*_*c*_, *y*_*c*_) according to (8). Without loss of generality, we assume that 1 ≤ *x*_*c*_, *y*_*c*_ < 2. Then, control points *ϕ*_*kl*_, for *k*, *l* = 0,1, 2,3, are calculated as [23]

(10) $$\begin{array}{c} \phi_{kl}=\frac{w_{kl}z_{c}}{\sum_{a=0}^{3}\sum_{b=0}^{3}w_{ab}^{2}}, \end{array}$$

where *w*_*kl*_ = *B*_*k*_(*s*)*B*_*l*_(*t*) and *s* = *x*_*c*_ − 1, *s* = *y*_*c*_ − 1. Now, for each data point in **P**, (10) can be used to calculate the set of 4 × 4 control points in its neighborhood. In cases where data points are sufficiently close, these neighborhoods may overlap and therefore they may assign different values to shared control points. Multiple assignments to a control point *ϕ* can be resolved by considering the data points in its 4 × 4 neighborhood. For each of these points, (10) gives *ϕ*_*ij*_ a different value *ϕ*_*c*_. To compromise among the different values, *ϕ*_*ij*_ is chosen to minimize error *e*(*ϕ*_*ij*_) = ∑_*c*_(*w*_*c*_ *ϕ*_*ij*_ − *w*_*c*_ *ϕ*_*c*_)^2^. The least-squared solution is

(11) $$\begin{array}{c} \phi_{ij}=\frac{\sum_{c}w_{c}^{2}\phi_{c}}{\sum_{c}w_{c}^{2}}. \end{array}$$

The shape of approximation function *f* could be directly affected by the density of control lattice Φ overlaid on domain *Ω*. Coarser Φ yields a smoother approximation at the expense of approximation accuracy while finer Φ enables **P** to be more closely approximated, although *f* will tend to contain local peaks near the data points. Thus, we have a tradeoff between the surface smoothness and accuracy of the approximation. Lee et al. [23] proposed a multilevel algorithm to avoid this tradeoff. This algorithm makes use of a hierarchy of control lattices Φ_*k*_ to generate a sequence of functions *f*_*k*_ whose sum approaches the desired approximation function, that is, *f* = ∑_*k*_ *f*_*k*_. This process can be optimized by using B-spline refinement to reduce the sum of the functions *f*_*k*_ into one equivalent B-spline function [23]. This optimization yields large computational savings.

Note that only the coarsest lattice Φ_0_ is calculated based on the original data point **P** and a rough approximation to data point is obtained using this control lattice. All consecutively finer lattices provide to approximate and eliminate the remaining error. Interpolation is achieved when sufficiently fine lattices are employed to remove all residuals. A sufficient condition for interpolation also has been presented in [23], which is based on the data distribution and the minimum distance among all pairs of data points.

**Figure 4:**
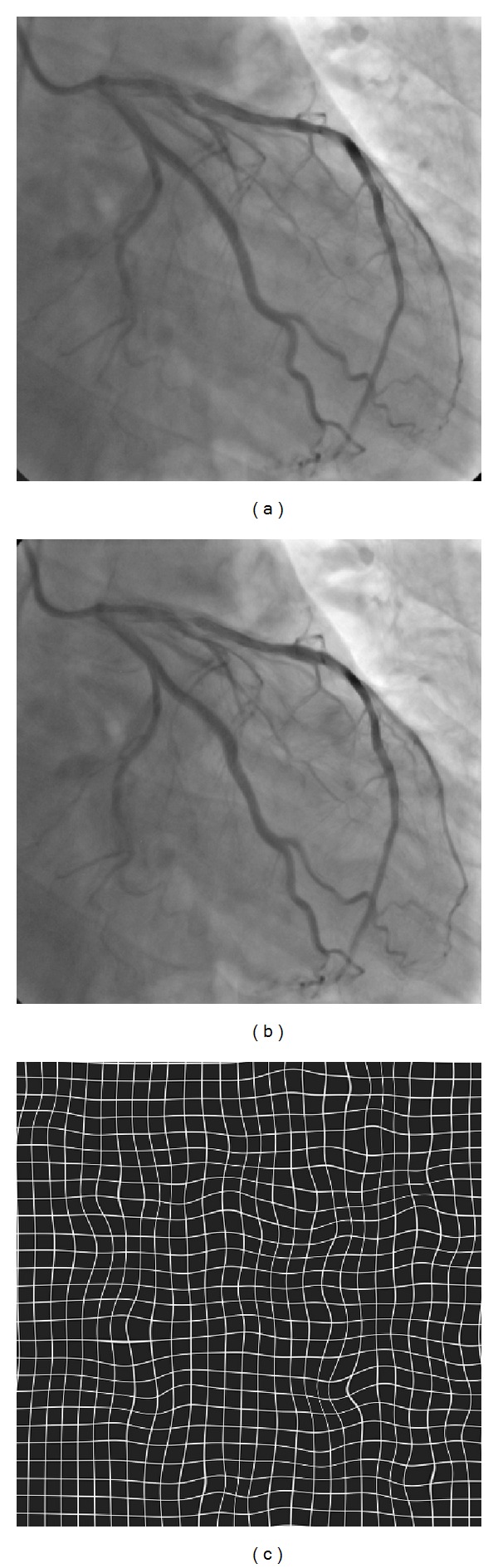
An elastic image warping example using multilevel B-spline interpolation. (a) Original image. (b) Warped image. (c) Warped test grid that shows the behavior of warp function.

Figure 4 shows an example of elastic image warping by means of multilevel B-spline interpolation. In this example, a warp function computed based on multilevel B-spline interpolation of a set of random control point pairs is applied to a coronary angiography image (Figure 4(a)). The warped coronary angiography image and a test grid deformed using the derived warp function are given in Figures 4(b) and 4(c), respectively. As can be seen in Figures 4(b) and 4(c), the multilevel B-spline image warping can produce locally smooth deformation which is demanded for image registration of coronary angiography images.

### 2.4. Grey-Level Distortion Reduction

Even if the correct dense correspondence between the mask and live images in a sequence has been recovered (in terms of a displacement vector field) and the mask image has been warped accordingly, the background of the resulting DSA images will not necessarily be totally homogeneous. Aside from noise, the main source of these artifacts is alterations in local densities that occur due to contractions and expansions of tissues. Other causes of gray-level distortion artifacts include fluctuations in the intensity of X-rays [25].

For reduction of gray-level distortion artifacts, we use a method based on gray-scale morphological operations. In this method, the so-called morphological *bottom-hat* transformation of mask and live images are subtracted from the original mask image and live image, respectively, to generate enhanced images:

(12) $$\begin{array}{c} \hat{M}=M-B_{\text{hat}}\left(M\right), \end{array}$$

(13) $$\begin{array}{c} \hat{L}=L-B_{\text{hat}}\left(L\right), \end{array}$$

where *B*_hat_(·) denotes the morphological bottom-hat transformation that for a gray-scale image *I* is defined as the closing of *I* minus *I*:

(14) $$\begin{array}{c} B_{\text{hat}}\left(I\right)=\left(I\cdot s\right)-I, \end{array}$$

where *s* is a structuring element. One of the principal applications of bottom-hat transformation is in removing dark objects on a light background by using a structuring element in the closing operation that does not fit the objects to be removed. The difference operation then yields an image in which only the removed components remain. Therefore, after subtracting the bottom-hat transformation of mask and live images from the original ones according to (12) and (13), the visibility of dark objects that structuring element does not fit them can be enhanced. In this paper, a nonflat, ball-shaped structuring element whose diameter in the *x*-*y* plane is larger than the largest expected vessel width is used.

**Figure 5:**
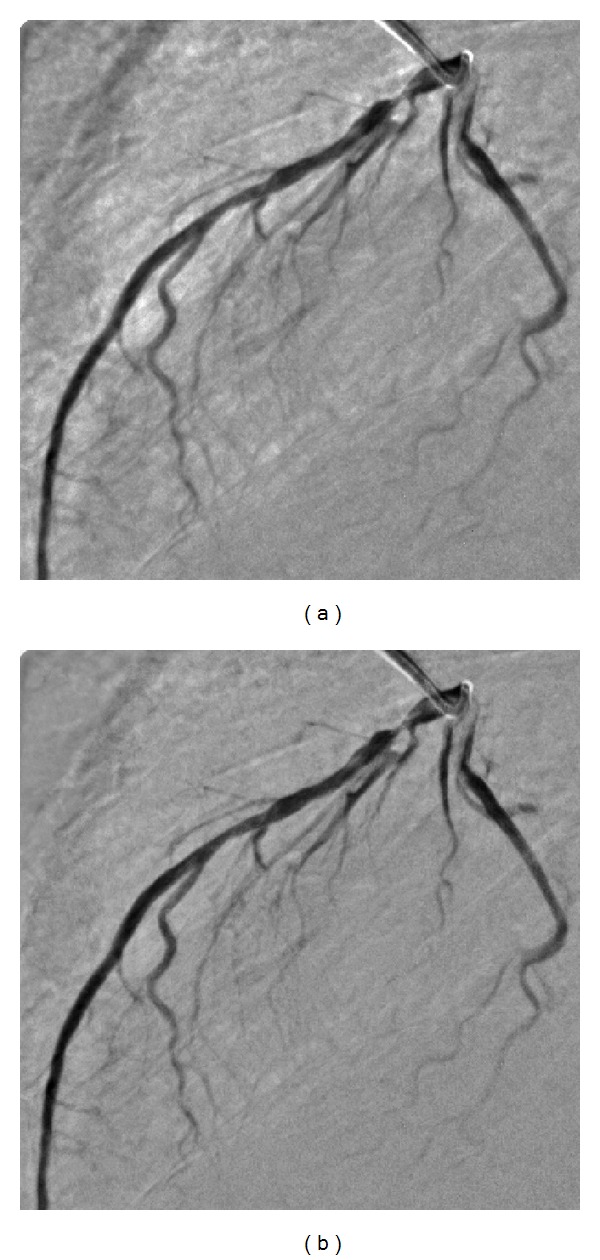
Reduction of gray-level distortion artifacts. (a) A DSA image resulted after registration of related mask and live images. Due to gray-level distortion, the background in this image is not entirely homogeneous. (b) Result of gray-level distortion artifacts reduction after application of the proposed approach.

We found from experiments that gray-level distortion artifacts in the background of DSA image resulted after registration can be reduced if the enhanced mask image $\hat{M}$ is warped in image warping step of registration algorithm instead of original mask image and then subtracted from enhanced live image $\hat{L}$. This results in more homogeneous background in DSA image. An example of gray-level distortion reduction based on this approach is shown in Figure 5.

## 3. Algorithm Overview

The algorithm here is a summary of the operations involved in the registration of two images of a digital angiographic image sequence, presented and discussed in the previous section. Given a mask image and a live image from an angiographic image sequence, the registration is accomplished by carrying out the following steps.

1. Using the proposed feature selection approach described in Section 2.1, select the set of control points *P* = {**p**_*i*_} in the live image *L* by following steps 1–7 of Section 2.1.
2. Given the set of control points *P*, determine the displacement of every selected control point **p**_*i*_ ∈ *P* by means of template-matching technique using entropy of histogram of differences as similarity measure and hill-climbing algorithm for optimization.
3. Using the morphological method presented in Section 2.4 generate the enhanced mask image $\hat{M}$ and enhanced live image $\hat{L}$ according to (12) and (13), respectively.
4. Given the displacements of control points **p**_*i*_, calculate the complete displacement vector field using multilevel B-spline interpolation and warp the enhanced mask image $\hat{M}$ based on this displacement vector field.

## 4. Experimental Results

The registration algorithm presented in previous sections was implemented in Matlab and was tested on AMD processor 2.3 GHz with 1 GB RAM. To evaluate the performance of the proposed algorithm to the reduction of motion artifacts in DSA images we carried out experiments on various data sets. All data sets which are used in the experiments are clinical coronary angiographic image sequences consisting of 512 × 512 images and are acquired from a Siemens AXIOM Artis FC DSA imaging system.

### 4.1. Quantitative Evaluation of Registration Results

For quantitative evaluation of proposed algorithm, it is first applied to register simulated images. Each test data set consists of a live image taken from a cardiac angiography image sequence and its elastically warped version as simulated live image. To generate the simulated live images, we used the similar approach as described in Section 2.2.1 by random selection of control points on live image, addition of an uncorrelated random shift to both *x* and *y* dimensions of control points, and then warping of live image according to generated control point pairs by means of multilevel B-spline interpolation. In this way, the true transformation parameters and therefore true correspondences between the two images are known. By calculating the root-mean-squares (RMS) error of the control point pairs after registration of simulated data, the algorithm can be evaluated objectively. The RMS error, *δ*_RMS_, is computed as

(15) $$\begin{array}{c} \delta_{\text{RMS}}={\left(\frac{1}{n}\left(\sum_{i=1}^{n}{||\left(x_{i}^{\prime },y_{i}^{\prime })-({\hat{x}}_{i},{\hat{y}}_{i}\right)||}^{2}\right)\right)}^{0.5}, \end{array}$$

where $\left({\hat{x}}_{i},{\hat{y}}_{i}\right),\;i=1,2,\ldots ,n$, are corresponding points of a set of *n* randomly selected control points on the simulated live image which are calculated using the known transformation, and (*x*_*i*_′, *y*_*i*_′),  *i* = 1,2,…, *n*, are corresponding points estimated after registration of the live and simulated live images. Moreover, the registration performance may be evaluated according to the RMS error reduction ratio that is defined as

(16) $$\begin{array}{c} R=\frac{\delta_{0}-\delta }{\delta_{0}}\%, \end{array}$$

where *δ*_0_ denotes the initial RMS error between the corresponding control points of the live and simulated images and *δ* is the value of RMS error after registration.

Note that the motions of the control points should not change the topology of the simulated image. Therefore, the minimum distance between randomly selected control points and maximum random shifts is strictly restricted such that the motion boundaries of control points do not have overlap with each other. Two experiments are conducted to compare the proposed registration algorithm versus different control point selection approaches and multiresolution/iterative registration algorithms. In these experiments, typically 30 control points pairs with *x* and *y* displacements ranging from −10 to 10 pixels are used to generate the simulated data. Other parameters of the algorithm were kept fixed to the values shown in Table 1.

#### 4.1.1. Comparison of Control Point Selection Approaches

This part is devised to compare different control point selection approaches used in our registration algorithm including edge-based scheme [2, 15], exclusion technique [11], and the proposed hybrid approach. In edge-based scheme, control points are extracted from important edges, and in the exclusion technique, control points are selected on a regular grid and then those points that are located in regions with insufficient contrast variation being excluded. 

We applied our registration algorithm with these three different control point selection approaches to register simulated images. Then, a set of corresponding point pairs were calculated using true transformation parameters of the simulated data. The RMS error between these points and the ones given after registration is used for evaluation of different control point selection strategies.  Table 2 gives the details of pixel errors, including maximum, minimum, and RMS errors after registration of the ten simulated image pairs. In average, the proposed hybrid selection method leads to more accurate results and the RMS error reduction ratio for the ten image pairs is more than other two methods.

#### 4.1.2. Comparison with Multiresolution and Iterative Algorithms

In this section, we compare the proposed registration method with two competing algorithms introduced, respectively, by Yang et al. [20] and Wang and Zhang [19] on the simulated data. The first one is a multiresolution registration algorithm in which the mask image is decomposed to coarse and fine subimage blocks iteratively and each block is rigidly registered to the live image. The calculated registration transform in each decomposition level is used as an initial guess for the next finer level. The second algorithm uses an iterative procedure in which a set of corresponding interest points are extracted from live and mask images for each iteration and registration transform represented by TPS is refined according to this correspondence.

Comparison of these three algorithms is accomplished according to registration accuracy and computational time. Similar to previous section, accuracy is evaluated based on RMS pixel error between true corresponding control points and ones estimated by registration of live and simulated live image pairs.  Table 3 lists the final registration results for the ten simulated image pairs. It can be seen from these results that the proposed registration algorithm has considerably lower computational time than other two ones. The average RMS error reduction ratio for the ten image pairs is 80.6% for proposed method that is better than the corresponding value for iterative refinement and multiresolution blocking algorithms. Note that the registering a pair of angiography images with proposed registration method is significantly faster than multiresolution blocking.

### 4.2. Registration Results on Clinical Images

**Figure 6:**
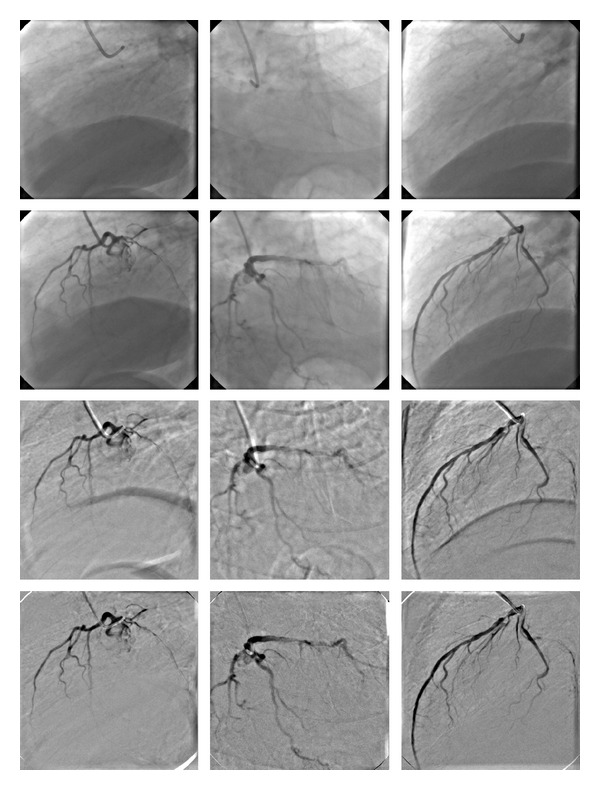
Application of the proposed method for the registration of coronary angiographic images. Rows from top to bottom correspond to mask images, live images, the original subtraction images (DSA images before registration), and the subtraction images after correction for motion artifacts, using the proposed registration technique.

We carried out several experiments on clinical data sets. The results of applying the proposed registration technique to the three cardiac angiographic image data sets are demonstrated in Figure 6. Rows from the top to bottom correspond to mask images, live images, the original subtraction of the mask images and the live images, and the subtraction after correction for motion artifacts, using the proposed registration approach. By comparing the original subtraction images to the registered ones, it can be seen that most of the motion artifacts have been disappeared after registration.

## 5. Discussion

From Figure 6 and by comparing DSA images before and after of image registration, it can be seen that the artifacts resulted by nonrigid motions have been significantly reduced after applying the proposed approach and, therefore, the quality of these images is improved. As it is shown by several authors [2, 15, 17], the complex and non-rigid motions cannot be corrected by applying the global pixel-shifting technique provided on standard DSA imaging systems. Moreover, in the case of coronary angiograms we deal with local and nonlinear motions of tissues such as respiratory and cardiac motion while the main motions in peripheral and cerebral angiography are global rigid motions induced by patients. So registration algorithms designed for peripheral and cerebral angiography which use rigid models as registration transform, are not suitable for coronary angiography images.

To cope with local differences between the mask and live images in coronary angiography and compensation of non-rigid motions, multiresolution block registration algorithm [20] and iterative TPS refinement [19] were proposed. However, these algorithms have high computational time which makes them not acceptable for real-time clinical applications. Computational efficiency is thus an important factor that must be considered. The results of experiments described in Section 4.1.2 indicate that our registration algorithm is effective and fast and outperforms alternative approaches, regarding both registration accuracy and required computation time.

As a final remark, it is important to note that there is a natural limitation of any registration algorithm for angiography images. These are in fact 2D projections of 3D anatomical structures and it is practically impossible to retrieve a 2D geometrical transformation from the projection images that completely represents the 3D motion of the original objects [25]. Specially, in the case of cardiac and abdominal angiography we are encountered with independently moving superimposed structures in some regions of the image. True correspondences between mask and live images might not be obtained in such regions, mainly because of neighborhood operations used in the matching process. This problem is another reason that the registration of cardiac angiography images is much harder and complicated than that of cerebral and peripheral angiography.

## 6. Conclusions

In this paper, a new approach to the fast registration of digital angiographic image sequences is proposed. The method involves the selection of a set of control points on the live image by using a hybrid approach based on an edge-detection scheme and Harris corner detector. The displacement of control points with respect to the mask image is computed by means of template matching, using the entropy of histogram of differences (ENT) as similarity measure. An objective evaluation method is developed in this paper on simulated data for comparison of different similarity measures and is demonstrated that ENT is the most suitable similarity measure for registration of angiography images. In order to reduce the computational cost in template matching procedure, an iterative hill-climbing approach is used for the optimization. For reducing the gray-level distortion artifacts in the background of subtraction image resulted after registration, a method based on gray-scale morphological operations is used to generate the enhanced mask and live images. The final correction is accomplished by elastic warping of the enhanced mask image using multilevel B-spline interpolation function.

To evaluate our method, we applied it to various clinical data sets. Registration results of our method with three different control point selection strategy and the results of two competing algorithms were evaluated on simulated data. The overall conclusion from the experimental results is that, in general, the proposed method is effective and very fast and has the capability to reduce the most of motion artifacts when applied to coronary angiography images.

## Figures and Tables

**Table 1:** Values of the parameters of the proposed registration algorithm.

Parameter	Description	Value
*σ*	Scale of edge-detection filter	1
*D*_min⁡_	Minimum distance between selected control points	25
*w*	Size of template used in template matching	51
*h*	Number of control lattices in hierarchy used in multilevel B-spline interpolation	7

**Table 2:** Comparison of registration results for three different control point selection approaches for ten simulated image pairs.

Image pairs	Initial error before registration	Error of the control point pairs after registration											
		Edge-based approach				Exclusion technique				Proposed hybrid approach			
	RMS (*δ*_0_)	Max	Min	RMS	(*δ*_0_ − *δ*)/*δ*_0_%	Max	Min	RMS	(*δ*_0_ − *δ*)/*δ*_0_%	Max	Min	RMS	(*δ*_0_ − *δ*)/*δ*_0_%
No. 1	6.098	13.115	0.103	2.861	53.1%	12.741	0.144	3.437	43.6%	7.591	0.048	1.486	75.6%
No. 2	6.892	10.090	0.030	3.192	53.7%	12.556	0.109	3.925	43.0%	3.097	0.045	0.881	87.2%
No. 3	6.442	12.071	0.042	2.642	59.0%	10.972	0.047	3.419	46.9%	7.378	0.109	1.302	79.8%
No. 4	6.201	10.548	0.102	2.711	56.3%	11.827	0.064	2.769	55.3%	3.818	0.008	1.061	82.9%
No. 5	6.363	9.792	0.164	2.781	56.3%	9.461	0.057	3.005	52.8%	5.673	0.058	1.289	79.8%
No. 6	6.270	7.903	0.162	2.242	64.2%	9.798	0.075	2.336	62.7%	2.934	0.035	0.960	84.7%
No. 7	6.813	9.606	0.066	3.312	51.4%	10.540	0.107	3.456	49.3%	5.497	0.037	1.614	76.3%
No. 8	6.920	12.286	0.079	3.389	51.0%	14.201	0.061	4.359	37.0%	3.666	0.064	1.229	82.2%
No. 9	7.399	11.843	0.013	4.152	43.9%	13.573	0.052	3.932	46.9%	5.925	0.085	1.490	79.9%
No. 10	7.494	15.460	0.051	5.423	27.6%	11.019	0.051	3.645	51.3%	7.293	0.011	1.643	78.1%

Average	—	—	—	—	51.6%	—	—	—	48.9%	—	—	—	**80.6%**

**Table 3:** Comparison of final registration results of the iterative refinement algorithm [19], the multiresolution blocking algorithm [20], and the proposed algorithm on ten simulated image pairs.

Image pairs	Initial error before registration	After registration								
		Iterative refinement			Multiresolution blocking			Proposed approach		
	RMS (*δ*_0_)	RMS	(*δ*_0_ − *δ*)/*δ*_0_%	Elapsed time (s)	RMS	(*δ*_0_ − *δ*)/*δ*_0_%	Elapsed time (s)	RMS	(*δ*_0_ − *δ*)/*δ*_0_%	Elapsed time (s)
No. 1	6.098	1.942	68.1%	268.8	1.511	75.2%	8954	1.486	75.6%	14.9
No. 2	6.892	1.396	79.7%	270.5	1.015	85.3%	9958	0.881	87.2%	13.5
No. 3	6.442	1.682	73.9%	269.8	0.870	86.5%	8745	1.302	79.8%	13.1
No. 4	6.201	1.191	80.8%	274.5	1.603	74.1%	9180	1.061	82.9%	15.6
No. 5	6.363	1.825	71.3%	254.8	1.254	80.3%	9553	1.289	79.8%	14.4
No. 6	6.270	1.258	79.9%	275.1	1.542	75.4%	9210	0.960	84.7%	12.4
No. 7	6.813	2.133	68.7%	296.3	1.532	77.5%	8768	1.614	76.3%	14.2
No. 8	6.920	1.349	80.5%	265.0	1.272	81.6%	9003	1.229	82.2%	14.3
No. 9	7.399	1.846	75.1%	289.6	1.784	75.9%	9548	1.490	79.9%	14.2
No. 10	7.494	2.408	67.9%	256.5	2.112	71.8%	9346	1.643	78.1%	15.1

Average	—	—	74.6%	272.1	—	78.4%	9226	—	**80.6%**	**14.2**

## References

[1] Brody WR (1982). Digital subtraction angiography. *IEEE Transactions on Nuclear Science*.

[2] Meijering EHW, Zuiderveld KJ, Viergever MA (1999). Image registration for digital subtraction angiography. *International Journal of Computer Vision*.

[3] Zitová B, Flusser J (2003). Image registration methods: a survey. *Image and Vision Computing*.

[4] Brown LG (1992). Survey of image registration techniques. *ACM Computing Surveys*.

[5] van den Elsen PA, Pol EJD, Viergever MA (1993). Medical image matching: a review with classification. *IEEE Engineering in Medicine and Biology Magazine*.

[6] Maintz JBA, Viergever MA (1998). A survey of medical image registration. *Medical Image Analysis*.

[7] Hajnal JV, Hill DLG, Hawkes DJ (2001). *Medical Image Registration*.

[8] Fitzpatrick JM, Pickens DR, Grefenstette JJ, Price RR, James AE (1987). Technique for automatic motion correction in digital subtraction angiography. *Optical Engineering*.

[9] Hua P, Fram I, Loew MH Feature-based image registration for digital subtraction angiography.

[10] Cox GS, de Jager G, Loew MH Automatic registration of temporal image pairs for digital subtraction angiography.

[11] Buzug TM, Weese J, Fassnacht C, Lorenz C, Hohne K-H, Kikinis R (1996). Using an entropy similarity measure to enhance the quality of DSA images with an algorithm based on template matching. *Visualization in Biomedical Computing*.

[12] Buzug TM, Weese J, Lorenz C, Beil W, Del Bimbo A (1997). Histogram-based image registration for digital subtraction angiography. *Image Analysis and Processing*.

[13] Buzug TM, Weese J (1998). Image registration for DSA quality enhancement. *Computerized Medical Imaging and Graphics*.

[14] Taleb N, Jetto L (1998). Image registration for applications in digital subtraction angiography. *Control Engineering Practice*.

[15] Bentoutou Y, Taleb N, Chikr El Mezouar M, Taleb M, Jetto L (2002). An invariant approach for image registration in digital subtraction angiography. *Pattern Recognition*.

[16] Cao Z, Liu X, Peng B, Moon Y-S DSA image registration based on multiscale Gabor filters and mutual information.

[17] Bentoutou Y, Taleb N (2005). Automatic extraction of control points for digital subtraction angiography image enhancement. *IEEE Transactions on Nuclear Science*.

[18] Bentoutou Y, Taleb N (2005). A 3-D space-time motion detection for an invariant image registration approach in digital subtraction angiography. *Computer Vision and Image Understanding*.

[19] Wang J, Zhang JQ An iterative refinement DSA image registration algorithm using structural image quality measure.

[20] Yang J, Wang Y, Tang S, Zhou S, Liu Y, Chen W (2007). Multiresolution elastic registration of X-ray angiography images using thin-plate spline. *IEEE Transactions on Nuclear Science*.

[21] Ping L, Hong N, Ye S An efficient method for image registration in DSA.

[22] Harris C, Stephens M A combined corner and edge detector.

[23] Lee S, Wolberg G, Shin SY (1997). Scattered data interpolation with multilevel b-splines. *IEEE Transactions on Visualization and Computer Graphics*.

[24] Canny J (1986). A computational approach to edge detection. *IEEE Transactions on Pattern Analysis and Machine Intelligence*.

[25] Meijering EHW, Niessen WJ, Viergever MA (1999). Retrospective motion correction in digital subtraction angiography: a review. *IEEE Transactions on Medical Imaging*.

[26] Powell MJD (1964). An efficient method for finding the minimum of a function of several variables without calculating derivatives. *Computer Journal*.

[27] Duchon J, Schempp W, Zeller K (1977). Splines minimizing rotation-invariant semi-norms in sobolev spaces. *Constructive Theory of Functions of Several Variables*.

[28] Bookstein FL (1992). Principal warps: thin-plate splines and the decomposition of deformations. *IEEE Transactions on Pattern Analysis and Machine Intelligence*.

